# Pathological Characteristics of Periodontal Disease in Patients with Chronic Kidney Disease and Kidney Transplantation

**DOI:** 10.3390/ijms20143413

**Published:** 2019-07-11

**Authors:** Mineaki Kitamura, Yasushi Mochizuki, Yasuyoshi Miyata, Yoko Obata, Kensuke Mitsunari, Tomohiro Matsuo, Kojiro Ohba, Hiroshi Mukae, Atsutoshi Yoshimura, Tomoya Nishino, Hideki Sakai

**Affiliations:** 1 Department of Nephrology, Nagasaki University Hospital, Nagasaki 852-8501, Japan; 2 Division of Blood Purification, Nagasaki University Hospital, Nagasaki 852-8501, Japan; 3 Department of Urology, Nagasaki University Graduate School of Biomedical Sciences, Nagasaki 852-8501, Japan; 4 Department of Respiratory Medicine, Unit of Basic Medical Sciences, Nagasaki University Graduate School of Biomedical Sciences, Nagasaki 852-8501, Japan; 5 Department of Periodontology and Endodontology, Nagasaki University Graduate School of Biomedical Sciences, Nagasaki 852-8501, Japan

**Keywords:** periodontal disease, chronic kidney disease, kidney transplantation, immunosuppressive therapy

## Abstract

Chronic kidney disease (CKD) is recognized as an irreversible reduction of functional nephrons and leads to an increased risk of various pathological conditions, including cardiovascular disease and neurological disorders, such as coronary artery calcification, hypertension, and stroke. In addition, CKD patients have impaired immunity against bacteria and viruses. Conversely, kidney transplantation (KT) is performed for patients with end-stage renal disease as a renal replacement therapy. Although kidney function is almost normalized by KT, immunosuppressive therapy is essential to maintain kidney allograft function and to prevent rejection. However, these patients are more susceptible to infection due to the immunosuppressive therapy required to maintain kidney allograft function. Thus, both CKD and KT present disadvantages in terms of suppression of immune function. Periodontal disease is defined as a chronic infection and inflammation of oral and periodontal tissues. Periodontal disease is characterized by the destruction of connective tissues of the periodontium and alveolar bone, which may lead to not only local symptoms but also systemic diseases, such as cardiovascular diseases, diabetes, liver disease, chronic obstructive pulmonary disease, and several types of cancer. In addition, the prevalence and severity of periodontal disease are significantly associated with mortality. Many researchers pay special attention to the pathological roles and clinical impact of periodontal disease in patients with CKD or KT. In this review, we provide information regarding important modulators of periodontal disease to better understand the relationship between periodontal disease and CKD and/or KT. Furthermore; we evaluate the impact of periodontal disease on various pathological conditions in patients with CKD and KT. Moreover, pathogens of periodontal disease common to CKD and KT are also discussed. Finally, we examine the importance of periodontal care in these patients. Thus, this review provides a comprehensive overview of the pathological roles and clinical significance of periodontal disease in patients with CKD and KT.

## 1. Introduction

Chronic kidney disease (CKD) is defined as either the presence of kidney damage, such as proteinuria and hematuria, or decreased kidney function (estimated glomerular filtration rate (eGFR) < 60 mL/min/1.73 m^2^ for >3 months) [1]. CKD is a crucial health issue, as it has a negative impact on prognosis and quality of life (QoL) due to the increasing risk of various pathological conditions including hypertension, diabetes, smoking, aging, autoimmune diseases, systemic inflammation, urinary tract infections, urinary stones, lower urinary tract obstruction, recovery from acute kidney injury, low birth weight, and drug toxicity [2,3,4,5]. Conversely, several risk factors of CKD, such as hypertension, diabetes, smoking, and aging have also been identified [1]. Thus, early recognition of CKD and the prevention of CKD progression are highly desirable. Furthermore, growing research has shown that periodontal disease and CKD are positively correlated, although periodontal disease is an important risk factor for CKD as it has the potential to be modified and treated. Several cohort studies have investigated the relationship between CKD and periodontal disease [6,7,8]. Systematic reviews have also reported that periodontal disease is associated with CKD [9,10,11]. Moreover, the severity of periodontal disease is correlated with a decline in kidney function [12]. Although periodontal disease is a mixed infection, gram-negative bacilli play a major role. *Porphyromonas gingivalis* is implicated in periodontal disease, and elevated levels of immunoglobulin G (IgG) antibodies against *P. gingivalis* were shown to positively correlate with the onset and progression of CKD [13]. Despite speculations that CKD is closely associated with the occurrence and progression of periodontal disease, detailed pathological characteristics at the molecular level and the clinical significance of periodontal disease in CKD patients are not fully understood and still remain unknown.

Renal replacement therapy is performed to maintain QoL and life in patients with end-stage renal disease (ESRD). As renal replacement therapy, peritoneal dialysis, hemodialysis, and kidney transplantation (KT) are usually selected according to the patient’s wishes; his/her clinical condition, the original disease, and/or the presence of transplant organ donor sources. In general, KT has some advantages in the improvement of life expectancy and QoL compared with dialytic therapies [14,15]. In fact, in contrast to peritoneal dialysis and hemodialysis, limitation of amount of drinking water and excessive dietary restriction are necessary the patients received KT when transplanted renal graft achieves normal function [16]. In addition, concentrations of uremic toxins in patients with KT are remarkably lower than those dialysis patients even though technology of dialysis is improving [17]. Such advantages are important to prevent the periodontal disease because oral health is improved by increased drinking of water and normalization of salivary properties [18,19]. Conversely, we also should note that kidney function in KT patients is usually equal to that of CKD patients because there may be still a functional contralateral kidney. Although the differences in renal function are limited between CKD and KT patients, KT should be considered an immunosuppressive status due to the immunosuppressive therapy required in these patients, and this immunoreactivity plays an important role in periodontal disease [20].

In this review, at first, we summarize information regarding the pathogenesis of periodontal disease to provide a better understanding of the specific pathological mechanisms of periodontal disease in CKD and KT patients. Briefly, we pay special attention to common pathological conditions in patients with periodontal diseases and in patients with CKD or KT. Based on this information, we then introduce the impact of periodontal disease on various other diseases and on the pathological status of CKD patients. Moreover, this review also reveals the clinical significance of periodontal disease in patients with KT. Specifically, we discuss the influence of immunosuppressive drugs used to treat oral pathological conditions including gingival overgrowth in patients with KT. Furthermore, we compare the periodontal pathogens implicated in CKD and KT patients. Finally, the impact of periodontal therapy on outcome of kidney function in these patients is discussed, and we comment on the importance of periodic oral care and appropriate periodontal treatment. Thus, this review provides comprehensive and cross-sectional information on the pathological roles of periodontal disease in CKD and KT, while we emphasize that a better understanding of pathogens, pathological roles, and the impact of immunosuppressive therapy is essential to maintain the QoL, avoid complications, and improve survival in these patients.

## 2. Modulators of Periodontal Disease

It is necessary to exacerbate inflammation in distant tissues for local inflammation of the teeth to injure distant organs. There are several mechanisms through which periodontal bacteria affect multiple organs, such as systemic bacteremia, cytokine release and inflammation, swallowing to the gastrointestinal tract, and direct exposure through alveolar bone destruction. The first two mechanisms are common pathways for multiple organ failure. Bacteremia caused by bacteria invades the blood stream through the periodontitis lesion and an influx of inflammation mediators, such as interleukin (IL)-6 and tumor necrosis factor (TNF)-α, produced in the lesion can be introduced into the systemic circulation. Importantly, the pathological significance of such processes in patients with CKD is different from that of healthy individuals. For example, case fatality rates at 30 and 90 days in ESRD patients with bloodstream infections was 15% and 25%, respectively; however, the rate in control patients was 0% [21]. In addition, a uremic environment due to CKD was reported to modulate the levels of various cytokines and inflammation-related molecules, including toll-like receptor in immune cells, leading to increased inflammation [22]. Furthermore, other investigators have paid special attention to the relationships between CKD and oxidative stress, endothelial dysfunction, and adhesion molecules [23,24,25]. However, these factors that may be modulated by CKD play important roles in inflammation under various physiological and pathological conditions [23,26,27,28]. Based on these considerations, we review previous reports describing the pathological significance of bacteremia, cytokines and inflammation-related molecules, oxidative stress, and endothelial dysfunction in periodontal disease.

### 2.1. Bacteremia

How do periodontal bacteria move via the blood stream and damage multiple organs? First, gingival ulceration in the periodontal pocket enables bacteria to enter the systemic circulation [29]. Recirculating leukocytes engulf and destroy foreign antigens in the immune response; however, some periodontal disease bacteria, such as *P. gingivalis* and *Actinobacillus actinomycetemcomitans*, can survive intracellularly, and may exploit macrophages and/or dendritic cells as vehicles, much like a Trojan horse, which causes silent systemic inflammation [29]. Although it remains unknown as to whether periodontal bacteria proliferate on blood vessel walls, components of periodontitis bacteria have been detected in arteriosclerotic lesions [30]. Thus, bacteremia is an important step in the progression to systematic inflammation in patients with periodontal diseases. In fact, blood bacteria levels were higher in patients with periodontitis than in those with gingivitis (*p* < 0.0001), and its level was positively associated with worse periodontal parameters [31].

### 2.2. Cytokines and Inflammation-Related Molecules

The biological activity observed in periodontal lesions has suggested that various cytokines are involved in the pathogenesis of periodontal disease [32]. Numerous cytokines are known to be associated with periodontal disease, and cytokines are categorized by their function. Representative inflammatory cytokines include IL-1, IL-6, IL-8, and TNF-α. These inflammatory cytokines enhance vascular permeability, which may enhance bacteremia and stimulate fibroblasts and inflammatory cells, which in turn induce other cytokines. They also enhance the expression of endothelial adhesion molecules, such as intercellular adhesion molecule (ICAM)-1 and vascular cell adhesion molecule (VCAM)-1, E-selectin, and chemokines such as monocyte chemoattractant protein (MCP)-1 and IL-8 [33]. In addition, inflammatory cytokines regulate bone resorption and inhibit bone formation, which are associated with the local expansion of periodontal lesions through alveolar bone loss [32] and systemically with CKD-mineral bone disorder (CKD-MBD). Fibroblast growth factor (FGF)-23, which has been suggested to have a central role in CKD-MBD and in the regulation of serum phosphorus levels, has been associated with higher inflammatory cytokine levels [34]. Moreover, inflammatory cytokines cause an expansion of the mesangial matrix or induce interstitial fibrosis [35]. If bacteremia does not cause direct damage through in situ inflammatory cytokine production in the targeted organ, increased inflammatory cytokines in local periodontal lesions cause systematic inflammation via the blood stream. Other important cytokines are growth factors, such as FGF, platelet-derived growth factor (PDGF), and transforming growth factor-β (TGF-β). Connective tissue growth factor (CTGF), which normally maintains tissue homeostasis, can induce fibrosis during the inflammatory response. Renal fibrosis has a negative impact on renal prognosis, especially in patients with CKD. Considering these findings, cytokines in periodontal diseases increase vascular permeability, enhance the expression of adhesion molecules, and cause up-regulation of TGF-β. These responses may be associated with proteinuria via glomerular permeability, renal thrombosis, and renal fibrosis, respectively, and result in a deterioration of renal function [35,36].

Periodontal disease, which is associated with systemic disorders, is associated with gram-negative organisms such as *P. gingivalis,* rather than gram-positive organisms. Generally, gram-positive cocci and rods are primarily detected in subgingival plaques of healthy people. However, in plaques formed in the gingival pocket of chronic periodontitis patients, gram-negative anaerobic bacilli increase resulting in the transition to bacterial flora, which cause the formation of more complex pathogenic plaques [37]. Lipopolysaccharides (LPS) derived from the periodontal pathogens will be delivered systemically in blood vessels to other organs. An increase in inflammation through Toll-like receptor 2 and/or 4 in the innate immune system will be observed in these organs [29]. The interaction between LPS and toll-like receptors is quite complicated: The toll-like receptor-mediated pathogenic action of LPS in the immune system differs depending on the derived pathogens and the toll-like receptors [38]. For example, LPS derived from *P. gingivalis* has been associated with inducing urinary protein via Toll-like receptor 2 of the renal glomerular vascular endothelial cells and the progression of kidney diseases via Toll-like receptor 4 signaling in a diabetic animal model [39]. Nonetheless, it should be noted that the downstream signaling of Toll-like receptors has crucial roles in inflammation [32].

### 2.3. Oxidative Stress

Reactive oxygen species (ROS) are an important primary defense factor in periodontal disease [40]. ROS are produced by inflammatory cells such as polymorphonuclear leukocytes and vascular smooth muscle cells, and nicotine adenine dinucleotide phosphate oxidase is a major source of ROS generation. However, although the main target of ROS is nuclear DNA, excessive ROS production will generate lipid peroxide through homeostasis of oxidative balance in tissues, and lipid peroxide in local periodontal lesions is associated with periodontal diseases [41]. Moreover, ROS are associated with oxidative stress and long-lasting systemic oxidative stress, which is thought to cause multi-organ failure [42]. Reactive nitrogen intermediates are also important in oxidative stress. For example, peroxynitrite, which is produced by nitric oxide and superoxide anion causes endothelial damage [43]. There are several oxidative stress markers such as malondialdehyde (MDA), 8-hydroxydeoxyguanosine (8-OHdG), and 4-hydroxy-2-nonenal (4-HNE). It is plausible that oxidative stress has a significant impact on local periodontal lesions, and the effects of oxidative stress on the systemic inflammation have been shown by several research groups. For example, tissue 8-OHdG levels increased in multiple organs, such as the liver, heart, kidneys, and brain in a periodontal inflammation model [41]. Furthermore, in saliva, MDA and 8-OHdG levels are thought to be associated with oxidative periodontal lesions, and 4-HNE levels in the saliva may reflect systemic inflammation [44]. Conversely, there has been a report indicating that nuclear factor erythroid 2-related factor (NrF2), which is a key regulator of antioxidants, plays an important role in protecting against tissue destruction in periodontitis [45]. Thus, the balance between oxidative stress and antioxidants is speculated to be associated with occurrence, severity, and progression of periodontal disease.

### 2.4. Inflammatory Reaction and Endothelial Dysfunction

One of the most important mechanisms in systemic organ dysfunction in periodontal disease is due to endothelial dysfunction, which is associated with platelet aggregation, foam-cell formation, and development of atheroma [46]. In CKD, proteinuria is one of the most important surrogate markers of kidney prognosis and reflects endothelial dysfunction [34]. As stated above, inflammatory cytokines originating from bacteremia or paracrine from distal periodontal lesions will cause vascular permeability and vascular wall injury [32]. In addition to inflammatory cytokines, endothelial adhesion molecules, such as ICAM-1 and VCAM-1, play an important role in vascular injury via the activation of endothelial cells and smooth muscle cell proliferation [47]. Endothelial injury will cause arterial stenosis, resulting in hypertension [47].

In addition to the above observations, an animal model showed that ridocaine inhibited endothelial dysfunction of the systematic artery in rats with periodontal inflammation and decreased ROS was associated with such a mechanism [48]. Furthermore, investigation in patients with chronic periodontitis demonstrated that endothelial dysfunction of the branchial artery occurred with greater frequency in patients with periodontitis than in those without periodontitis [46]. Interestingly, this study and other investigators showed that together with various cytokines and inflammation-related molecules, oxidative stress regulated the relationship between periodontitis and endothelial dysfunction [49,50].

### 2.5. Matrix Metalloproteinases and Transglutaminases

When discussing the pathological mechanisms and development of periodontal disease, information regarding gingival remodeling and healing steps is important. In addition, an understanding of the protective system of oral tissues from microbial challenge in periodontal disease is also essential. From this standpoint, there is an interesting study that focused on the pathological roles of matrix metalloproteinase (MMP) and transglutaminases in patients with chronic periodontitis [51]. In short, when MMP-2 and -9 and transglutaminase-1–3 were analyzed in 22 patients with chronic periodontitis and healthy controls, transglutaminase-1 and 3 mRNA levels in chronic periodontitis patients were lower than those in healthy controls [51]. Finally, this study showed that different transglutaminases might regulate gingival remodeling/healing and adaptive processes in patients with chronic periodontitis. Conversely, it has also been reported that transglutaminase-2 may play an important role in vascular calcification in CKD [52]. Thus, the pathological roles of transglutaminases in CKD are not fully understood.

## 3. Periodontal Disease and Chronic Kidney Disease

### 3.1. Impact on Periodontal Disease from other Pathological Conditions

As discussed above, there is evidence suggesting that there is a direct relationship between the inflammation of periodontal disease and CKD, and renal function may decline for various reasons. CKD may evolve from any chronic renal disorder etiology, and typically includes diabetes, hypertension, and chronic nephritis [1,4]. In contrast, periodontal diseases can cause dysfunction of various organs, such as the heart, liver, and kidney. Therefore, the reduction in renal function due to the burden of other diseases as a result of periodontal disease should be considered.

#### 3.1.1. Diabetes

The most important complication of periodontal disease is diabetes, and the risk of diabetes mellitus is increased by periodontal disease, suggesting that this association is bidirectional. In fact, the prevalence of diabetes is higher in patients with periodontal disease than in those without periodontitis [53]. Furthermore, the Hisayama study in Japan showed that patients who developed glucose intolerance were more likely to have periodontal disease than the group that did not develop glucose intolerance [54]. Diabetic nephropathy with overt proteinuria occurs as a result of long-term diabetes, but diabetes patients with periodontal disease have a higher risk of cardiovascular disease compared with patients without periodontal disease [55]. Therefore, diabetic nephropathy caused by periodontal disease shortens the clinical course of ESRD [56]. With regard to the molecular mechanisms involved, the inflammatory cytokines IL-1 and IL-6, TNF-α; fibrotic growth factors TGF-β and CTGF; and oxidative stress have all been implicated in the original pathogenesis of diabetic nephropathy [57]. There is a close relationship between diabetes and periodontal disease and glycemic control is crucial, not only for kidney function, but also periodontal disease. Leukocyte function is poorly controlled in diabetic patients and this may worsen and exacerbate periodontal disease [58]. The effects of periodontal disease on diabetic nephropathy vary depended upon the patients’ condition. Thus, diabetic patients with periodontal disease should be treated intensively both for glycemic control and periodontal diseases.

#### 3.1.2. Hypertension

Hypertension is one of the most important risk factors of premature cardiovascular disease, including CKD. A systematic review has shown the relationship between periodontal disease and hypertension [59]. As stated above, inflammation in response to bacteremia or inflammatory mediators from periodontal lesions plays a role in hypertension. Possible pathophysiological mechanisms in periodontal disease and high blood pressure are endothelial dysfunction, reduction of nitric oxide bioavailability, oxidative stress, renin-angiotensin-aldosterone system activation, arterial stiffness, and atherosclerosis [47]. Conversely, cross-sectional clinical studies have shown that risk factors for CKD in patients with periodontal disease include diabetes [60,61] and hypertension [60].

#### 3.1.3. Liver Diseases

Periodontal disease is considered to be associated with different forms of liver disease, one of which is nonalcoholic fatty liver disease (s). The correlation between IgG antibody titers against periodontal pathogens has been shown to be increased in non-alcoholic fatty liver disease (NAFLD) patients [62]. Similar to injuries in other organs, the mechanisms involved in periodontal diseases and NAFLD are described as follows: LPS derived from periodontopathic bacteria are transferred to the blood and act directly on the liver, or LPS-derived inflammatory cytokines act indirectly on the liver [63]. NAFLD can affect multiple extrahepatic organs, such as the cardiovascular system, and increases the risk of developing CKD, with insulin resistance being an important mediating factor [64].

#### 3.1.4. Others

In recent years, nutritional status in kidney disease has attracted increasing attention and patients with CKD are facing protein energy wasting (PEW) complications [59]. Since proinflammatory cytokines affect the brain and cause anorexia, persistent inflammation is associated with PEW [65]. CKD is a very complicated disease, and the etiologies of inflammation observed in patients with CKD are multifactorial. Although any form of inflammation can cause PEW, periodontal disease is a potential etiology for persistent inflammation [66]. Periodontal pathogens that are swallowed and reach the intestine cause changes in the intestinal microbiota, resulting in a situation resembling metabolic bacteremia such as that found in obesity. Although the direct effect of periodontal pathogens on the gut microbiota is controversial, oral microbiota could also alter the gut microbiota [67]. Recent studies have revealed that not only diabetes and obesity but also CKD is closely associated with the intestinal flora [68]. Dysbiosis of the gut microbiota is believed to be associated with periodontal disease, and administration of *P. gingivalis* was shown to alter the gut microbiota and affect multiple organs in an animal model [69]. Thus, CKD should be considered a multifactorial disease; it is extremely difficult to identify specific etiological factors, but it should be taken into consideration that periodontal disease indirectly causes renal dysfunction.

**Figure 1:**
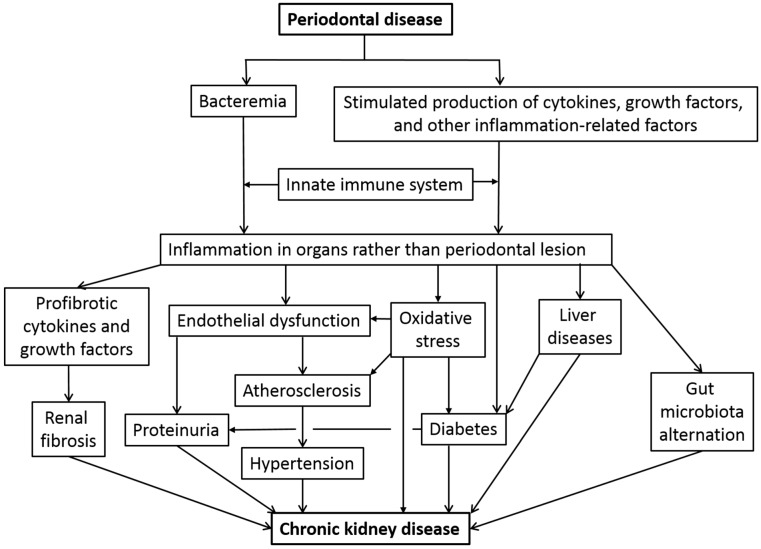
Impact of periodontal disease in the pathogenesis in chronic kidney disease.

A scheme illustrating the complex mechanisms involved in periodontal disease and CKD is shown in Figure 1.

### 3.2. Periodontal Therapy and CKD Patients with Periodontal Disease

There is a bidirectional relationship between periodontal disease and CKD, and between periodontal disease and diabetes [70]. The results of this study still remain unclear, although if CKD proceeds and renal function deteriorates, lymphocyte function and monocyte/macrophage function will be impaired and the immune system will aggregate; consequently, the risk of infection will increase compared to the healthy population [4]. When it comes to CKD-MBD, hyperphosphatemia increases phosphate levels in saliva, which is associated with a higher risk of inflammation of the periodontium. Furthermore, higher levels of phosphorus itself are linked to systemic inflammation [71]. This will enhance the association of periodontal disease and CKD. Although periodontal disease is a crucial risk factor for the onset of kidney disease and progression of renal failure, it is a treatable and modifiable risk factor. eGFR might improve in patients with CKD, however patients with ESRD cannot improve their eGFR.

The treatment of periodontal disease attenuates systemic inflammation and improves surrogate markers of endothelial function [72]. Through the reduction of inflammatory cytokines, renal function was shown to improve after treatment of periodontal disease [73]. A systematic review also showed a favorable effect of periodontal treatment on eGFR [74]. Other reports have indicated that treatment for periodontal disease decreases asymmetric dimethylarginine [75] and 4-hydroxy-2-nonenal, surrogate markers of systemic inflammation [44] and endothelial function [9], respectively.

In a discussion on periodontal therapy in patients with CKD, we must also take into consideration the influence of immunosuppressive agents. In short, a variety of CKD, such as lupus nephritis and IgA nephropathy, is commonly treated with immunosuppressive agents [76,77]. Undoubtedly, the immune response is closely associated with infection control in almost organs and tissues including oral and periodontal tissues. Therefore, in CKD patients treated with immunosuppressive agents, a treatment strategy for periodontal disease must be planned according to its severity, renal function, and response to anti-bacterial agents. In addition, in some patients, decreasing exposure to immunosuppressive agents should also be discussed. Unfortunately, however, there is little information regarding appropriate dosage in these patients. We emphasize the importance of further studies on appropriate dosage and types of immunosuppressive agents to be used in CKD patients with severe periodontal disease, especially in non-responders to anti-bacterial agents.

## 4. Periodontal Disease and Kidney Transplantation

As mentioned above, KT is one of the major renal replacement approaches to maintain QoL in patients with ESRD, and KT might be the choice of renal replacement therapy to achieve normalized kidney function, which undeniably leads to both social and physical advantages for the patient [14,15]. However, immunosuppressive therapy is essential for maintenance of kidney allograft function to prevent rejection and renal dysfunction, although it may be the cause of several infectious diseases including periodontitis. In addition, there is a general agreement that immunosuppressive conditions sometimes lead to the progression of local infections to general bacteremia. In Japan a total of 1742 KT including 1544 from living donors (LD) and 198 from deceased donors (DD) were performed in the proportion of patients with ABO blood type, incompatible with KT and requiring desensitization for antibody removal before KT, was as large as 27.7% of KT from LD, while the dialysis period before KT was as long as 15.1 years from DD in a Japanese survey [78]. ABO incompatibility and exposure of long-term dialysis therapy lead to susceptibility to immunosuppression, which leads to the risk of developing infectious diseases including periodontal disease. It is important to understand the underlying biological mechanisms of periodontal disease and the immunological status of KT recipients. Moreover, it is important to identify an approach to treat periodontal diseases for the management of KT.

### 4.1. The Screening of Periodontal Disease Before and After KT

Screening for infectious diseases is important in the preparation of KT. It is well-known that poor oral health is common among CKD patients [79]. KT candidates are considered to have a high prevalence of periodontal disease due to the status of end-stage of renal insufficiency, which is called stage-five CKD. There has been a recent increase in the number of preemptive kidney transplantation (PEKT) procedures, which are defined as transplant before initiation of maintenance dialysis therapy, for the avoidance of complication by dialysis therapy [80]. PEKT is often performed at the progression of renal deterioration in the pre-dialysis stage. Moreover, the progressed renal insufficiency may occasionally fall into a severe immunocompromised state. Oral infections should be treated completely before exposure to immunosuppression for KT candidates, since recent potent immunosuppressive therapy regimens might lead to unexpected onset of infection including periodontitis. Oral health has been reported to be better during the KT period than at the pre-dialysis stage, and it thus it is important to treat oral infectious foci at the pre-dialysis stage in order to prevent adverse outcomes after KT [81]. However, lower salivary flow rates and higher numbers of drugs at the KT stage might influence the clinical outcome [81]. Patients with diabetic nephropathy associate with worse periodontal health and higher oral inflammatory conditions at the pre-dialysis stage to the same degree as the pre-transplant stage [82]. Due to the high prevalence of cardiovascular disease and requirements of extracorporeal circulation for hemodialysis, anti-coagulant therapy is used in many KT candidates. The surgical treatment of oral diseases in this condition may lead to active bleeding and cause general bacteremia or fatal sepsis. Preconditioning and necessary treatment are important for the KT recipient before and after exposure to immunosuppressive therapy. Finally, it should be considered that appropriate timing of treatment must be provided to KT recipients depending on the patient’s general status, CKD stage, and original cause of renal insufficiency [81,82].

### 4.2. Periodontal Condition and Clinical Outcome

Several investigators have examined the effects of periodontal conditions in relation to clinical outcome following KT. The studies reported a higher [83,84], similar [85], or lower [86] incidence of periodontitis in transplant recipients than in healthy controls. A limitation to the study results is that these differ depending on the definition of occurrence of periodontitis used. Ioannidou et al. [87] reported that none of the continuous periodontal variables were significantly associated with deterioration of allograft function due to the presence of strict criteria such as HLA-matching or history of acute rejection. A systematic review described the associations between periodontal status and clinical outcomes in KT recipients. A patient’s periodontal status might be associated with a larger left ventricular mass, greater carotid thickness, graft rejection, lower graft survival, and higher mortality rate in early KT periods among KT recipients [88]. These studies suggest that periodontal status may affect clinical outcomes of patient survival and graft survival due to the occurrence of cardiovascular disease and abnormal immunological responses with essential immunosuppressive therapy [86,87,88].

### 4.3. Influence of Immunosuppressive Drugs

Recently, potent strong immunosuppressive therapy is becoming more commonly used to prevent acute rejection, which has improved outcomes of KT in the modern era [89]. A protocol using everolimus (ERL) is often performed in addition to a standard protocol consisting of mycophenolate mofetil (MMF), tacrolimus (Tac), and corticosteroid (CS), which has allowed further improvements in treatment outcomes of KT [90,91]. However, protocols using multiple agents can lead to a condition of excessive immunosuppression, which may worsen oral status. There are currently different immunosuppressive agents available and it is clinically important to understand differences in each drug relative to the possible occurrence of periodontal disease. Calcineurin inhibitors (CNI) such as Cyclosporin A (CsA) and Tac have played a central role in immunosuppressive therapy of KT since the 1980s. CsA is characterized by a major side effect of gingival overgrowth (GO), which may lead to occasionally worsening of oral health. CsA-induced gingival enlargement has been reported to vary from 7–80% in different transplant centers [92]. Conversely, the use of ERL frequently results in adverse events of stomatitis and compromised status, and so it may be important to properly manage the oral environment during treatment [93]. However, few reports have examined the risk of developing periodontitis and other oral diseases in patients using ERL. Pereira-Lopes et al. compared the oral health status of KT recipients receiving Tac or ERL as immunosuppressants. The study showed that KT recipients receiving the ERL protocol presented reduced periodontal inflammation in comparison with patients receiving Tac [94]. In the study, patients receiving ERL were older and they experienced more limited periodontal inflammation. There might be a bias in the patient selection in the study. ERL is used for CNI minimization or corticosteroid elimination due to protection from CNI nephrotoxicity or various side effects of steroid therapy, which might affect long-term graft survival or patient survival [95,96,97]. Tac has similar adverse effects as CsA because both drugs share the same pharmacological mechanism of calcineurin inhibition. Several investigators have examined the occurrence of GO with the use of Tac, which has been less frequent than the use of CsA [98,99]. In a clinical study comparing exposure to CsA and Tac, it was reported that GO occurred later in the group using Tac, with the severity of GO in the Tac group being lower than that in the CA group [100]. Another study including a post-hoc analysis revealed that the prevalence of GO was 60.0% for CsA, 28.9% for Tac, and 15.6% for sirolimus, which was used as an mTOR inhibitor for the same purpose of ERL [101]. Tac may be an alternative agent to CsA in attempting to avoid adverse effects of GO [102,103]. Tac seems advantageous with regard to periodontal effects when using CNI as a standard immunosuppression protocol.

### 4.4. Immunosuppressant-Induced Gingival Overgrowth and Periodontal Condition

Gingival enlargement is commonly observed as a side effect of several different types of drugs, including anticonvulsants, calcium channel blockers, and immunosuppressants [92]. As previously mentioned, GO is common adverse effect of CsA usage, which may lead to the worsening of periodontal conditions in KT recipients. A multitude of factors may affect clinical and histopathological manifestations of immunosuppressant-induced GO. Several investigators have examined the causes of CsA-induced GO, which might associate with periodontal disease. The results of several studies examining specific factors found in the gingival crevicular fluid (GCF) were indicative of CsA-induced GO. Increased LL-37 peptide levels in the GCF is observed at the CsA-induced GO positive site with neutrophil infiltration and extended inflammation. LL-37 is an antimicrobial peptide and an important defense molecule of the host immune response [104]. Gürkan et al. [105] examined the association of many types of cytokine families with CsA-induced GO by the examination of the GCF. TGF-β_1_, whose levels are associated with clinical periodontal parameters, might be an exclusive mediator of CsA- or Tac-induced GO. Increased IL-6 and oncostain M under CsA usage have been reported to be involved in regulating the severity of inflammation and presence of GO, but cytokines of the IL-6 family might not be directly involved in the biological mechanisms associated with CsA-induced GO [106]. A study examining transgultaminase (TGM)-2, which had been shown to play a role in fibrosis by extracellular matrix accumulation, showed that TGM-2 might contribute to CsA-induced GO by modifying the GCF and plasma levels of oxidative stress markers [107].

The investigation of molecules contained in the GCF may provide researchers with a variety of insights associated with medication-induced GO. Conversely, several study groups have attempted to elucidate the mechanism of GO by investigating gene polymorphisms. IL-10 gene polymorphism might be associated with CsA-induced GO in KT recipients. A special genotype and allele might indicate an association with susceptibility to GO [108]. Alpha-2 integrin gene polymorphisms were not associated with CsA-induced GO but were associated with CsA-independent GO in KT recipients [109]. Interestingly, the length of the CAG repeat of the androgen receptor gene might link to CsA-induced GO via the analysis of polymorphisms [110]. Thus, many studies have been attempted to elucidate the mechanisms involved in drug-induced GO [104,105,106,107,108,109,110], but the biological mechanism of the gingival tissue response involved in immunosuppressant-induced GO is still not fully understood. Since gingival enlargement is directly associated with QoL, it is necessary to elucidate the causes of drug-induced GO by further scientific research and the challenge is to modify present immunosuppressive therapy regimens to avoid GO.

### 4.5. Inflammatory Markers of Periodontitis in KT

Inflammation may be associated with the deterioration of solid-organ function in transplantation recipients. Systemic inflammation occasionally originates from periodontal inflammation. There is clinical evidence that periodontal inflammation is linked to the occurrence of different systemic diseases including ESRD [111]. Moreover, the chronic inflammation of kidney allograft is often modulated by alloimmune-dependent mechanisms. The main cause of inflammation in solid-organ transplantation is dependent on human leukocyte antigen (HLA) mismatches or panel reactive antibody (PRA) scores, which may lead to acute humoral rejection [112,113]. Several inflammatory markers such as IL-6 and C-reactive protein (CRP) may be predictors of renal allograft survival associated with alloimmune-dependent acute rejection [114,115]. Moreover, specific cells associated with periodontal disease may produce significantly higher levels of IL-6, and serum CRP was reported to be elevated in patients with periodontal inflammation [116]. Shaqman et al. [117] compared periodontal disease and systemic inflammatory status of transplant recipients and age-matched controls, which led to the conclusion of the absence of any significant predictors of systemic inflammation in the population. In another study Blach et al. [118] examined several inflammatory markers such as TNF-α, IL-6, and high-sensitive CRP (hs-CRP) in KT recipients. The study demonstrated that severe chronic periodontitis was associated with increased serum hs-CRP, but not with any significant elevation of TNF- α or CRP. Elevated hs-CRP levels appeared to influence mortality after KT [118]; thus, it is important to monitor the levels of inflammatory markers such as hs-CRP, which may lead to the early detection and early treatment of periodontal disease. Furthermore, it would be more useful if an inflammatory marker specific to KT recipients undergoing immunosuppressive therapy were identified in real world studies.

## 5. Periodontal Pathogens in Chronic Kidney Disease and Kidney Transplantation

Several investigators have reported an increased frequency of periodontal pathogens in CKD and KT patients. There is general agreement that periodontal disease occurred by mixed infection, but not by a single pathogen alone [119,120]. With regard to CKD, Bastos et al. [121] reported that the frequencies of *P. gingivalis* and *Candida albicans* in pre-dialysis CKD patients showed a higher trend than the control group (94.7 versus 72.2% and 52.0% versus 26.3%, respectively); however, such difference did not reach statistical significance (*p* = 0.078 and *p* = 0.079, respectively). Conversely, the frequencies of *P. gingivalis* and *Treponema denticola* (*T. denticola*) were significantly associated with clinical detectable levels (*p* = 0.008 and *p* = 0.013, respectively) in CKD patients with periodontitis [121]. Conversely, other investigators have shown that *T. denticola*, *Tanerella forsythia* (*T. forsythia*), and *Parvimonas micra* (*P. micra*) were significantly associated with periodontal disease in patients with CKD [122]. In addition, the authors also found that *T. forsythia* was independently associated with periodontal disease in a multivariate analysis model including other significant pathogens, age, and estimated glomerular filtration rate (*p* = 0.008) [122].

Regarding KT patients, the frequency of *Streptococcus constellatus* in plaque samples form subjects with alveolar bone loss (36.4%) was significantly higher (*p* = 0.019) than that in samples without alveolar bone loss (3.7%) [83]. Conversely, a report also indicated that total counts of micro-organisms were increased between day 0 and day 90 after renal transplantation and between day 30 and day 90 after surgery [123]. In addition, the same study also showed that the frequency of β-hemolytic Streptococcus on day 90 after surgery (28.6%) was significantly lower (*p* = 0.031) than that on day 30 (44.4%) in KT patients with GO, but not in patients without GO [123]. Thus, in KT patients, quantitative and qualitative changes of microorganisms in the subgingival plaque might occur 90 days after surgery, and GO had an effect on expression of these microorganisms [117]. Unfortunately, there is limited information on the pathological significance of pathogens in patients with KT.

Conversely, a recent study evaluated the relationships between immunosuppressive agents and periodontal pathogenic bacteria in patients following solid organ transplantation [124]. Although it should be noted that the study population included three different types of organs (kidney, liver, and lung), the study demonstrated that changes in the levels of periodontal pathogenic bacteria dependent on immunosuppressive agents. In short, the prevalence of *P. micra*, was associated with immunosuppression exclusively with glucocorticoids; however, *P. gingivalis* was associated with combined immunosuppression of glucocorticoids, MMF, and Tac [124]. A summary is shown in Table 1.

## 6. Care of Periodontal Conditions in Chronic Kidney Disease and Kidney Transplantation

Many investigators pay special attention to the prevalence and severity of periodontal disease. In addition, the importance of periodontal treatment has been reported in CKD and KT patients because they may exhibit periodontal conditions worse than that of the healthy general population [124,125]. Currently, several pilot studies support this opinion in these patient groups [75,126]. Unfortunately, CRF or KT patients do not have much interest in screening and/or treatment of periodontal disease. Although we have no data in CRF and KT, there is a report that approximately 70% of hemodialysis facilities have no associated dental clinic [127]. There is an opinion that immunosuppressive therapy is not associated with the necessity of dental and periodontal treatment in patients with transplantation [124]. However, there is no general consensus for KT patients. Conversely, other investigators have suggested the importance of appropriate oral health in KT patients because oral hygiene status was closely associated with the development and degree of GO [128]. Finally, we also emphasize that periodic oral care and appropriate periodontal treatment with dentists are important to maintain QoL, inhibit the complications, and prolong the survival periods in CKD and KT patients. In recent years, the usefulness of various treatment strategies for improvement of oral health has been analyzed [129,130]. In addition, there is the opinion that clinical conditions, such as lipidemia in obesity, affect preventive and treatment strategies of chronic periodontitis [131]. Based on these facts, further detailed studies with larger study populations, longer observation periods, and analyses including broader periodontal disease-related factors are necessary to be able to reach definitive conclusions.

## 7. Conclusions

In this review, we introduced the pathological significance of periodontal diseases in patients with CKD. Specifically, we showed the increased risks of various pathological conditions, such as diabetes, hypertension, atherosclerosis, liver diseases, and gut microbiota alternation in these patients. Furthermore, in addition to the pathological roles of periodontal diseases in KT patients, we focused on the influence of immunosuppressive drugs on oral health, such as periodontitis, periodontal inflammation, and gingival enlargement. Moreover, the pathological significance and levels of pathogens were compared between patients with CKD and those with KT. Finally, we emphasized the importance of care of oral and periodontal condition to maintain the QoL, prevent complications, and improve survival in patients with CKD and KT. Conversely, a discussion regarding comprehensive preventive strategies of oral health, information on genetic polymorphisms and DNA methylation of oral diseases-related molecules is essential [132]. Based on such information, we suggest that further detailed prospective studies with larger populations and analyses at the molecular level should be performed to clarify the importance of oral health in these patients.

## Figures and Tables

**Table 1:** Pathological significance and levels of pathogens according to kidney status.

Pathogens	Status	Pathological Significance and Level of Pathogens	Ref.
*β-hemolytic Streptococcus*	KT	Associated with gingival overgrowth after transplantation	[123]
*Capnocytophaga spec*	KT	Lower compared to HD	[120]
*Enterococcus faecalis*	CKD	Higher compared to control	[125]
*Fusobacterium nucleatum*	KT	Lower in immunosuppression with glucocorticoid and mycophenolate	[124]
*Parvimonas micra*	CRF	Correlation with periodontal disease in multi-variate analysis model	[122]
	KT	Lower compared to HD	[122]
	SOT	Higher in immunosuppression with glucocorticoid	[124]
*Porphyromonas* *gingivalis*	CRF	Positively associated with clinical attachment level	[122]
	SOT	Lower immunosuppression with glucocorticoid, mycophenolate, and tacrolimus	[124]
*P* *revotella nigrescens*	CRF	Higher compared to control	[125]
*Streptococcus constellatus*	KT	Lower in subjects with peritoneal destruction	[83]
*Tanerella forsythia*	CKD	Correlation with periodontal disease in a multi-variate analysis model	[122]
	SOT	Lower in immunosuppression with glucocorticoid and mycophenolate	[124]
*Treponema denticola*	CRF	Positively associated with clinical attachment level	[121]
	CKD	Associated with periodontal disease	[122]

CKD—chronic kidney disease, KT—kidney transplantation, SOT—solid organ transplantation, and ref—reference.
